# Uncorking wine yeast genomics from grape to glass

**DOI:** 10.1093/femsyr/foag025

**Published:** 2026-06-03

**Authors:** Dariusz R Kutyna, Sylvie Dequin, Amparo Querol, Isak S Pretorius

**Affiliations:** The Australian Wine Research Institute, PO Box 46, Glenside SA 5065, Adelaide, Australia; Unité Mixte de Recherches Sciences pour lŒnologie, SPO, Univ Montpellier, INRAE, Institut Agro, 34060 Montpellier Cedex 2, France; Instituto de Agroquímica y Tecnología de los Alimentos, CSIC, C./ Catedrático Agustín Escardino, 7 - 46980 Paterna, Valencia, Spain; The Chancellery and ARC Centre of Excellence in Synthetic Biology, Macquarie University, Sydney, NSW 2109, Australia

**Keywords:** wine yeast biotechnology, synthetic yeast genomics, Sc 2.0, Yeast 2.0

## Abstract

Unravelling the genomic blueprint of a reference laboratory strain of the yeast *Saccharomyces cerevisiae* 30 years ago opened a new era in understanding yeast biology. Since then, genomics has transformed our ability to study, adapt, improve, and tailor wine yeast strains in the laboratory and manage them in the cellar. This minireview highlights key advances in wine yeast genomics, from early whole-genome sequencing of industrial *S. cerevisiae* strains to the recent assembly of complex non-*Saccharomyces* genomes, including the wine spoilage yeast *Brettanomyces bruxellensis*. Comparative genomics has revealed the genetic foundations of strain specific traits critical to fermentation performance, aroma production, stress tolerance, and microbial interactions in the vineyard and winery. Beyond cataloguing gene content, integrative genomic approaches have elucidated evolutionary dynamics, domestication events, and adaptation to industrial environments. These insights underpin the rational development of novel starter cultures and biotechnological interventions, fostering consistent wine quality and diversity of sensory profiles for targeted consumer markets. Looking ahead, advances in pan-genomics and functional genomics promise to deepen our understanding of metabolic networks, gene-environment interactions, and the broader ecological context of wine fermentation. Collectively, the study of wine yeast genomics not only illuminates fundamental biological principles but also provides practical tools for innovation, including pathway engineering with synthetic enzyme fusions, and the creation of purpose-built synthetic neo-chromosomes. Excitingly, *S. cerevisiae*, the first eukaryote to have its genome sequenced, is now poised to become the first eukaryote with an entirely synthetic genome – the Sc2.0 project – heralding a bold future for yeast genomics.

## Introduction

Wine fermentation is a highly complex bio ecological process, characterized by extensive variability of outcomes, which originates not only, but predominantly from the yeast strains driving alcoholic fermentation (Marsit and Dequin 2015, Pretorius 2016, Ruiz et al. 2023). *Saccharomyces cerevisiae* is a single-celled fungus, well recognized as a key domesticated player related to wine quality. This yeast in particular displays genomic signatures of domestication and adaptation that distinguish it from other ecological groups (Legras et al. 2007, Borneman et al. 2011, Pretorius 2017a). Despite the long history of human association with winemaking, reaching as far in the past as 8000 years based on some scientific reports (McGovern et al. 2017, Harutyunyan and Malfeito-Ferreira 2022), modern winemakers still face unpredictability in fermentation kinetics, stress tolerance, and aroma compound production. This underlines the substantial phenotypic diversity that exists among commercial and natural wine yeasts (Swiegers et al. 2005, Bisson 2019, Gardner et al. 2023). Also, as climate fluctuations result in alteration of grape composition; particularly sugar, nitrogen, and acid balance, and as grape producers increasingly seek consistency with fewer chemical interventions, the need for more predictable and precise fermentation has become especially desirable (van Leeuwen and Darriet 2016, Gardner et al. 2023).

Through extensive scientific developments in yeast genomics over the past 30 years, our understanding of laboratory and wine yeast biology has dramatically improved. High-throughput sequencing, population genomics, and functional genomic tools have revealed a wide range of genetic diversity among commercial as well as wild occurring yeast strains. The most obvious differences appear in domestication signatures, introgressions, and structural variations that shape key ecological traits (Borneman et al. 2011, Marsit and Dequin 2015). This growing body of knowledge has shifted winemaking practices that once relied on intuitive strain selection toward more targeted, genotype-informed approaches (Ambroset et al. 2011, Pretorius 2016). However, even with the abundance of genomic data now available, linking genotype to phenotype in a way that can reliably predict fermentation outcomes remains a major challenge. Many desirable traits, from nitrogen utilization efficiency to volatile compound biosynthesis, are polygenic, environment dependent, and shaped by interactions within the complex wine microbiome (Peter et al. 2018, Bisson 2019).

This review examines how genomic knowledge can improve our ability to address the unpredictability of wine fermentation in a more targeted and controllable manner. It highlights current understanding of wine yeast genomic diversity (Borneman et al. 2011, Peter et al. 2018, de Celis et al. 2024), recent progress in mapping the genetic bases of ecological traits (Ambroset et al. 2011, Gardner et al. 2023), and emerging models that integrate genomic data with systems level analyses and applications aiming at improving the predictability of winemaking outcomes (Bisson 2019). It also discusses the limitations that still hinder genotype to phenotype predictions and outlines opportunities for future research, including pan genomics, hybrid genomics, synthetic biology including the international *Synthetic Yeast Genome* (Yeast 2.0/Sc2.0) project (Pretorius and Boeke 2018, Williams et al. 2023, Erpf et al. 2025, Goold et al. 2025), and machine learning based trait prediction (Pretorius 2016, Peter et al. 2018, Barbosa et al. 2022). By bringing these advances together, the review aims to clarify how genomics can support more reliable, data-driven winemaking and where the field needs to advance to fully utilize the current potential and knowledge emerging from advances in wine yeast genomics.

## Historical milestones in the genomics of laboratory, wine, and other industrial yeasts

### Sequencing of laboratory strains and the first eukaryotic genome

The first major milestone in yeast genomics, and a defining moment for eukaryotic genome biology, was sequencing the genomes of *S. cerevisiae* laboratory strains genomes during the late 1980s and 1990s. Before whole-genome sequencing, yeast genetics relied on classical mutagenesis, tetrad analysis, and gene-by-gene experimental cloning. The *European Yeast Genome Sequencing* project transformed this landscape by coordinating more than 600 researchers across >90 laboratories worldwide, ultimately producing the first complete genome sequence of a *S. cerevisiae* reference strain S288c in 1996 (Goffeau et al. 1996). The project required approximately eight years of sustained international collaboration and cost an estimated USD 30 – 40 million, reflecting the labour- and cost-intensive nature of Sanger sequencing and the distributed chromosome-by-chromosome strategy used at the time (Dujon 1996, Goffeau et al. 1996, Oliver 1996). The resulting 12-Mb genome, organized into 16 chromosomes and encoding roughly 6000 genes, became the first fully sequenced eukaryotic genome. It provided a foundational scaffold for functional genomics, enabling systematic gene deletion collections (Giaever et al. 2002), large scale transcriptome profiling, and powerful comparative analyses that rapidly expanded the scope of yeast biology.

### Recognition of laboratory domestication and the limits of the S288c reference strain

Early sequencing efforts revealed the extent to which laboratory domestication had shaped the genomes of widely used research strains. Variants such as S288c, W303, and Σ1278b, often assumed to be phenotypically equivalent, were shown to differ substantially in auxotrophies, regulatory alleles, mitochondrial stability, and stress response loci (Ruderfer et al. 2006, Schacherer et al. 2007). Such genomic divergence had unexpected, yet practical consequences; phenotypes attributed broadly to ‘yeast biology’ were artifacts associated with specific laboratory lineages. As sequencing technologies advanced, it became clear that laboratory strains represented only a narrow subset of *S. cerevisiae* genomic diversity, prompting a shift toward sequencing genomes of more widely isolated, industrially-used strains, including strains that are typical for winemaking (Liti et al. 2009). Nonetheless, these early laboratory genome sequences established the conceptual and technical foundation for yeast genomics and remain the universal reference against which all subsequent wine yeast genome analyses were, and still are, interpreted.

### Emergence of industrial and wine yeast genomics

The transition from laboratory to industrial and wine yeast genomics marked the next major milestone. By the early 2000s, comparative genomic hybridization, BAC libraries, and partial sequencing projects had revealed that industrial strains differed markedly from the laboratory reference strains, such as S288c. They often carried extensive chromosomal rearrangements, aneuploidies, and introgressed regions from other *Saccharomyces* species (de Barros Lopes et al. 2002, Dunn et al. 2005). These early insights ignited curiosity within the scientific community and set the stage for the first complete genome of wine yeast strains, *S. cerevisiae* AWRI 1631 (Borneman et al. 2008) and EC1118 (Novo et al. 2009). The EC1118 genome revealed that wine yeasts were not simply wild isolates adapted to fermentation, but highly domesticated organisms with mosaic genomes shaped by human-driven selection. Notably, EC1118 carried several horizontally acquired genomic regions, including the well characterized ‘region C’, encoding functions related to nitrogen utilization, sugar transport, and stress tolerance that were absent from S288c (Novo et al. 2009, Marsit and Dequin 2015). These discoveries fundamentally shifted the understanding of wine yeast biology, highlighting the roles of horizontal gene transfer, interspecific hybridization, and structural genome variation in shaping industrial fermentation phenotypes.

### Population genomics, hybridization, and the long-read revolution

The publication of the first population-scale *S. cerevisiae* genomes marked another major turning point in yeast genomics. Large comparative studies, beginning with the sequencing of 36 diverse isolates in 2009, revealed the global population structure of *S. cerevisiae*, including distinct domestication lineages associated with wine, beer, saké, and bread fermentations (Liti et al. 2009). These analyses demonstrated that wine yeasts formed an undeniable unique clade, shaped by long-term association with human driven selection. This clade was characterized by reduced heterozygosity, specific metabolic adaptations, and signatures of bottlenecks linked to viticulture and oenology practices.

The rapid adoption of next generation sequencing technologies in the early 2010s further accelerated the field of yeast genomics, enabling high-throughput sequencing of hundreds of industrial strains and uncovering pervasive aneuploidy, copy number variation, and sub-telomeric plasticity as key drivers of fermentation performance. During the same period, genome sequencing revealed that many industrial yeasts were not pure *S. cerevisiae* lineages but interspecific hybrids and polyploids involving *S. kudriavzevii, S. uvarum*, and other *Saccharomyces* species (Gonzalez et al. 2006, Peris et al. 2017). These hybrid genomes provided evolutionary explanations for important oenological traits such as cold tolerance, glycerol yields, and the production of different aroma compounds.

**Figure 1:**
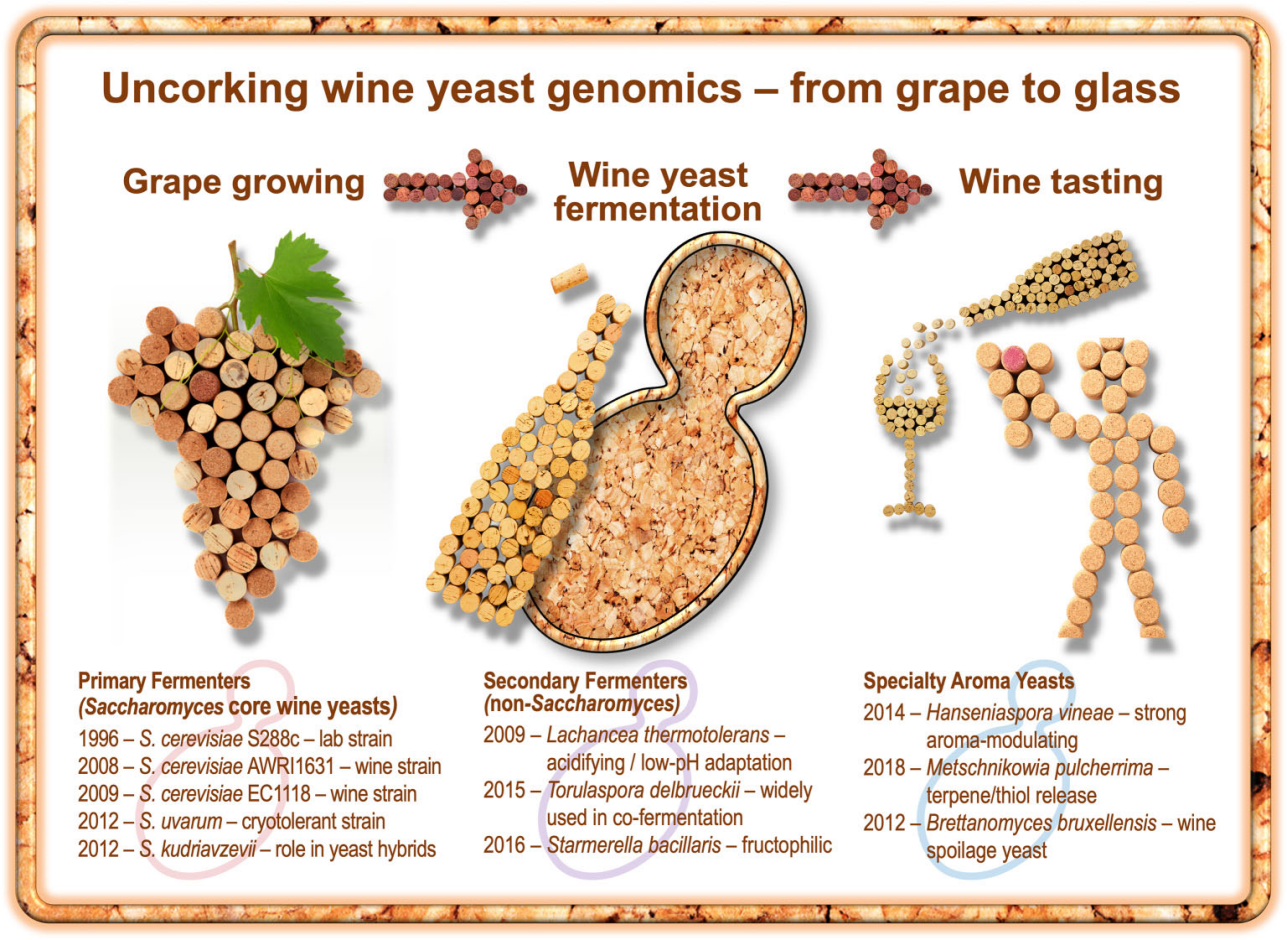
Uncorking wine yeast genomics—from grape to glass. Schematic overview of the wine production continuum, from grape growing through wine yeast fermentation to wine tasting, alongside major milestones in wine yeast genomics. The timeline highlights genome sequencing milestones for primary fermenters (core *Saccharomyces* wine yeasts), secondary fermenters (commercial non-*Saccharomyces* species), and specialty aroma yeasts. Listed species include *S. cerevisiae* strains S288c, AWRI1631, and EC1118, *Saccharomyces uvarum, Saccharomyces kudriavzevii, Lachancea thermotolerans, Torulaspora delbrueckii, Starmerella bacillaris, Hanseniaspora vineae, Metschnikowia pulcherrima*, and *Brettanomyces bruxellensis*, illustrating the expansion of genomic resources for yeasts relevant to fermentation performance, aroma modulation, and wine spoilage.

The arrival of long-read sequencing technologies in the late 2010s further transformed the field by enabling chromosome level assemblies. These assemblies resolved previously intractable sub-telomeric regions, structural variants, and mobile genetic elements; genomic compartments central to fermentation-related adaptation (Istace et al. 2017, Yue et al. 2017). These advances revealed extensive structural complexity in industrial strains, including large segmental duplications, translocations, and lineage-specific expansions of gene families involved in nitrogen metabolism, sugar transport, and stress tolerance (Giannakou et al. 2020, Gorkovskiy and Verstrepen 2021, Albillos-Arenal et al. 2025). Together, population genomics, high-throughput sequencing, and long-read assemblies provided an unprecedented view of industrial yeast genome evolution, showing that domestication is driven not only by point mutations and introgressions, but also significantly by large-scale structural variation and dynamic genome reorganization. This knowledge opened new possibilities for advances in yeast research and genomics (Pretorius 2022). Major milestones in the development of wine yeast genomics, from the laboratory reference strain to industrially relevant *Saccharomyces* and non-*Saccharomyces* species, are summarized in Fig. 1.

## Advances in genomic approaches applied to wine yeasts

### Population genomics, domestication, and structural variation

Large-scale genomic studies showed that wine yeasts form a distinct domesticated lineage within *S. cerevisiae* sensu stricto (Peter et al. 2018, Loegler et al. 2024). Centuries of human selection for reliable fermentation performance, stress tolerance, and positive sensory attributes have left clear marks on their genomes. Early comparative work showed that wine strains often have lower heterozygosity and selective sweeps around genes associated with nitrogen metabolism, sugar uptake, and stress responses (Peter et al. 2018, Legras et al. 2018), adaptations that gave them selective advantage in the nutrient poor, high sugar, ethanol accumulating environment of grape must fermentation. This is further supported by phenotypic evidence that wine-adapted strains exhibit a markedly stronger inherent ability to consume nitrogen during fermentation, contributing to their overall domestication and reliable performance in oenological conditions (Brice et al. 2018).

One of the most apparent features of wine yeast domestication is the widespread copy number variation (CNV) in genes linked to nutrient scavenging and stress resistance. Expansions in nitrogen transporters like *GAP1, PUT4*, and the *MEP* family help yeast to acquire restricted forms of nitrogen while growing under low yeast assimilable nitrogen (YAN) conditions, commonly found in many musts (Marsit and Dequin 2015). Duplications in heat shock protein genes improve tolerance to osmotic and ethanol stress. These CNVs frequently go hand-in-hand with chromosomal aneuploidies; recurrent extra copies of chromosomes III, VIII, and XV have been associated with faster fermentation and better sulfite tolerance (Legras et al. 2018, Peter et al. 2018).

The big population datasets, especially the 1 011-genome project (Peter et al. 2018), reveal that wine strains cluster tightly with low recombination, consistent with long periods of clonal propagation in winery isolation settings. This limited gene flow has helped fix beneficial structural variants, such as the well-known *SSU1* promoter rearrangement (translocation between chromosomes XV and XVI), which boosts sulfite efflux, confers increased sulfite tolerance, and shortens lag phases under sulfite stress (Pérez-Ortín et al. 2002). Other studies have highlighted rearrangements like this as some of the clearest genomic fingerprints of wine yeast domestication, driven by the routine use of sulfites in modern winemaking (Marsit and Dequin 2015).

**Figure 2:**
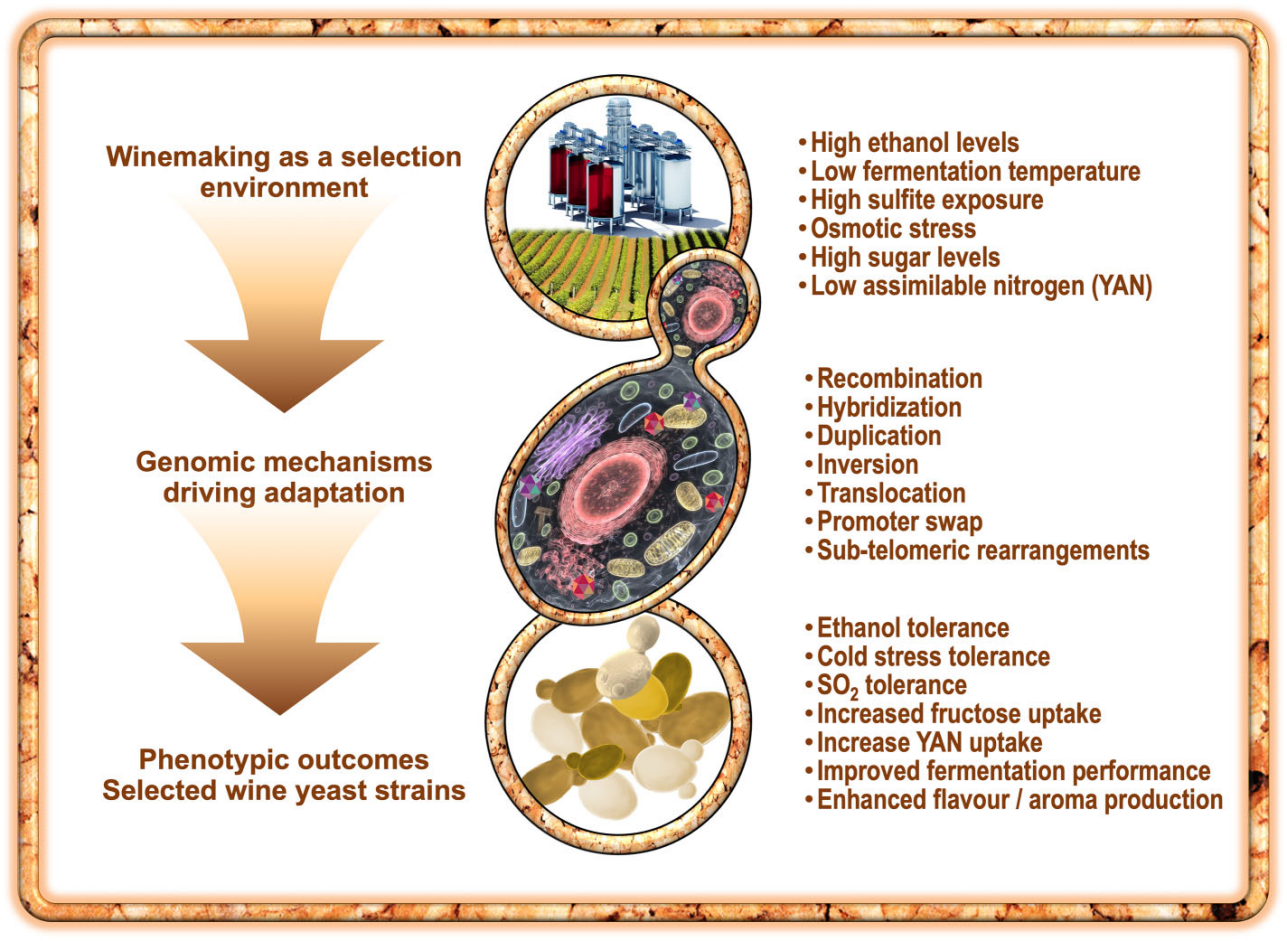
Genomic routes to adaptation and diversification in wine yeasts. Schematic representation of winemaking as a selective environment shaping wine yeast evolution. Environmental pressures associated with fermentation, including high ethanol, low temperature, sulphite exposure, osmotic stress, high sugar, and low yeast assimilable nitrogen (YAN), drive genomic mechanisms such as recombination, hybridization, duplication, inversion, translocation, promoter swapping, and subtelomeric rearrangements. These adaptive processes give rise to selected wine yeast strains with desirable phenotypic traits, including ethanol tolerance, cold resistance, SO₂ tolerance, improved fructose and YAN uptake, enhanced fermentation performance, and increased flavour and aroma production.

Wine yeast domestication is not a single historical event but rather an ongoing, multi-layered process influenced by both natural environment-driven selective pressures, and long-term human management in winemaking. Industrial strains continue to accumulate a variety of structural changes, such as segmental duplications, aneuploidies, and sub-telomeric rearrangements, that fine-tune key fermentation traits including ethanol tolerance, volatile ester production, and efficient nitrogen utilization (Pretorius 2016, Legras et al. 2018, Peter et al. 2018). These genomic adjustments are largely responsible for the consistent and robust performance of widely used commercial strains, such as EC1118, VIN13, or Montrachet, many of which carry multiple copy number variations (CNVs), horizontal gene transfers (HGT), and chromosomal amplifications. The examples of major selection pressures imposed by winemaking practices, together with the genomic mechanisms and phenotypic consequences that underpin wine yeast domestication and diversification, are summarized in Fig. 2.

### Introgressions, hybridization, and adaptive evolution

Introgression and hybridization have become two of the most powerful evolutionary forces shaping the genomes of wine yeasts. Comparative genomics shows that many *S. cerevisiae* wine strains carry introgressed DNA regions from closely related *Saccharomyces* species, most commonly *S. paradoxus, S. kudriavzevii*, and *S. uvarum*. These foreign regions often contain genes involved in cold tolerance, nitrogen metabolism, or sugar transport, giving wine yeasts clear adaptive advantages in the stressful, fluctuating environment of grape must fermentation (Marsit and Dequin 2015). Interestingly, many of these introgressed haplotypes show reduced recombination, which helps preserve favourable allele combinations and allows them to persist over long periods in wine yeast populations (Peris et al. 2012, Marsit and Dequin 2015). A classic and well-studied example of introgression in *S. cerevisiae* wine yeast is the widely used commercial strain EC1118. Its genome contains three distinct horizontally acquired regions (commonly labelled A, B, and C) that originated from non-*Saccharomyces* donors (Novo et al. 2009). These regions bring several industrially valuable functions; a high affinity fructose transporter (*FSY1*) that improves late-stage fructose uptake and helps prevent sluggish or stuck fermentations (Galeote et al 2010); oligopeptide transporters (*FOT1* and *FOT2*) that enhance nitrogen scavenging when the level of yeast assimilable nitrogen (YAN) is low (Marsit et al. 2015); and genes linked to flocculation, general stress tolerance, and sulfite resistance. Together, these acquired genes help explain why EC1118 performs so reliably across a wide range of winemaking conditions. These findings highlight EC1118 as a compelling example of how horizontal gene transfer (HGT) and introgression can rapidly expand the metabolic toolkit of industrial yeasts, often achieving in a few events what would take much longer through point mutations and gradual domestication alone (Pretorius 2016).

Hybridization has also played a major role in wine yeast evolution. Natural interspecific hybrids, particularly between *S. cerevisiae* and *S. kudriavzevii*, are frequently found in cooler wine regions. These hybrids combine the strong ethanol tolerance and fermentation robustness of *S. cerevisiae* with the superior cold tolerance of *S. kudriavzevii*, enabling efficient fermentations at lower temperatures (Gonzalez et al. 2006). Further, researchers have shown that these hybrids exhibit subgenome-specific gene expression patterns, with *S. kudriavzevii* alleles often preferentially expressed under cold conditions, demonstrating how hybrid genomes can flexibly tackle metabolic and physiological requirements in response to changing environmental conditions (Gonzalez et al. 2006, Marsit and Dequin 2015). Other hybridization-driven adaptations are also well documented. For instance, *S. cerevisiae* × *S. uvarum* hybrids, which are widely used in sparkling wine production, are able to utilize malic acid more effectively and perform better at low temperatures (Jolly et al. 2014). Introgressed alleles from *S. paradoxus* are also linked to improved trehalose metabolism, enhancing stress survival during the later, more challenging stages of fermentation (Liti et al. 2009, Marsit and Dequin 2015). These examples illustrate how hybridization serves as a powerful mechanism for genome scale innovation, allowing wine yeasts to rapidly acquire complex, multi-gene traits, which would be very difficult to evolve through incremental mutational steps alone (Pretorius 2016).

Overall, introgression, horizontal gene transfer, and hybridization reveal the deeply mosaic nature of wine yeast genomes. Adaptive evolution in these strains is driven not just by classical domestication within *S. cerevisiae*, but by ongoing genetic exchange across species boundaries. These processes are largely responsible for the genomic diversity and phenotypic flexibility that characterize modern wine yeasts.

### Functional genomics and trait mapping for fermentation performance

Functional genomics has greatly improved our understanding of how genetic variation drives fermentation phenotypes in wine yeasts. Early Quantitative Trait Loci (QTL) mapping studies, mostly using wine and laboratory strain crosses, have shown that major oenological traits, like nitrogen utilization, aroma production, and stress tolerance, are polygenic and involve many small effect alleles, frequent epistasis, and strong genotype-environment interactions (Ambroset et al. 2011, Cubillos et al. 2011, Peltier et al. 2019). A notable example is the identification of QTLs influencing stuck or sluggish fermentations, where natural allelic variation was found to interact with ethanol and temperature stress (Peltier et al. 2019). Examples also include allelic variation in *GAT1* and *MEP2* affecting ammonium uptake and nitrogen catabolite repression, polymorphisms in *IRC7* controlling the release of volatile thiols from cysteinylated precursors (a key contributor to the aroma of Sauvignon Blanc and similar styles) (Roncoroni et al. 2011), as well as QTL analyses of volatile compound production, revealing a complex polygenic architecture with numerous loci influencing esters, higher alcohols, and central carbon metabolites, highlighting tight genetic links between aroma formation and core metabolic pathways (Eder et al. 2018), and multi-locus interactions underlying ethanol tolerance, with genes such as *SSD1, HSP12*, and *TPS1*/*2* contributing to osmotic and ethanol stress resistance. This polygenic architecture also explains why fermentation performance is highly sensitive to must composition, temperature, and nutrient availability, all of which can substantially alter the expression of the underlying genetic networks (Pretorius 2016).

Transcriptomics and proteomics studies have revealed how gene expression evolves dynamically during wine fermentation, with wine strains typically upregulating stress response, sugar transporter, and aroma biosynthesis genes as the process progresses (Marks et al. 2008, Rossouw et al. 2012). Notably, alcohol acetyltransferases *ATF1* and *ATF2* are strongly induced mid-fermentation and control acetate ester production, which gives many wines their characteristic fruity character. Furthermore, nitrogen limitation triggers a broad metabolic reprogramming, increased flux through the Ehrlich pathway (leading to higher alcohols biosynthesis), modified redox balance (affecting glycerol levels), and altered fatty acid metabolism (influencing ester profiles) (Rossouw et al. 2012, Walker et al. 2014). Particularly important contributions have been made to understanding these nitrogen dependent responses, demonstrating that low nitrogen coordinates changes in central carbon metabolism, amino acid biosynthesis, and stress pathways; changes that directly shape fermentation kinetics and aroma outcomes (Crepin et al. 2017). These findings have practical implications, supporting the development of nitrogen-responsive biomarkers and improved nutrient supplementation strategies in winemaking.

**Figure 3:**
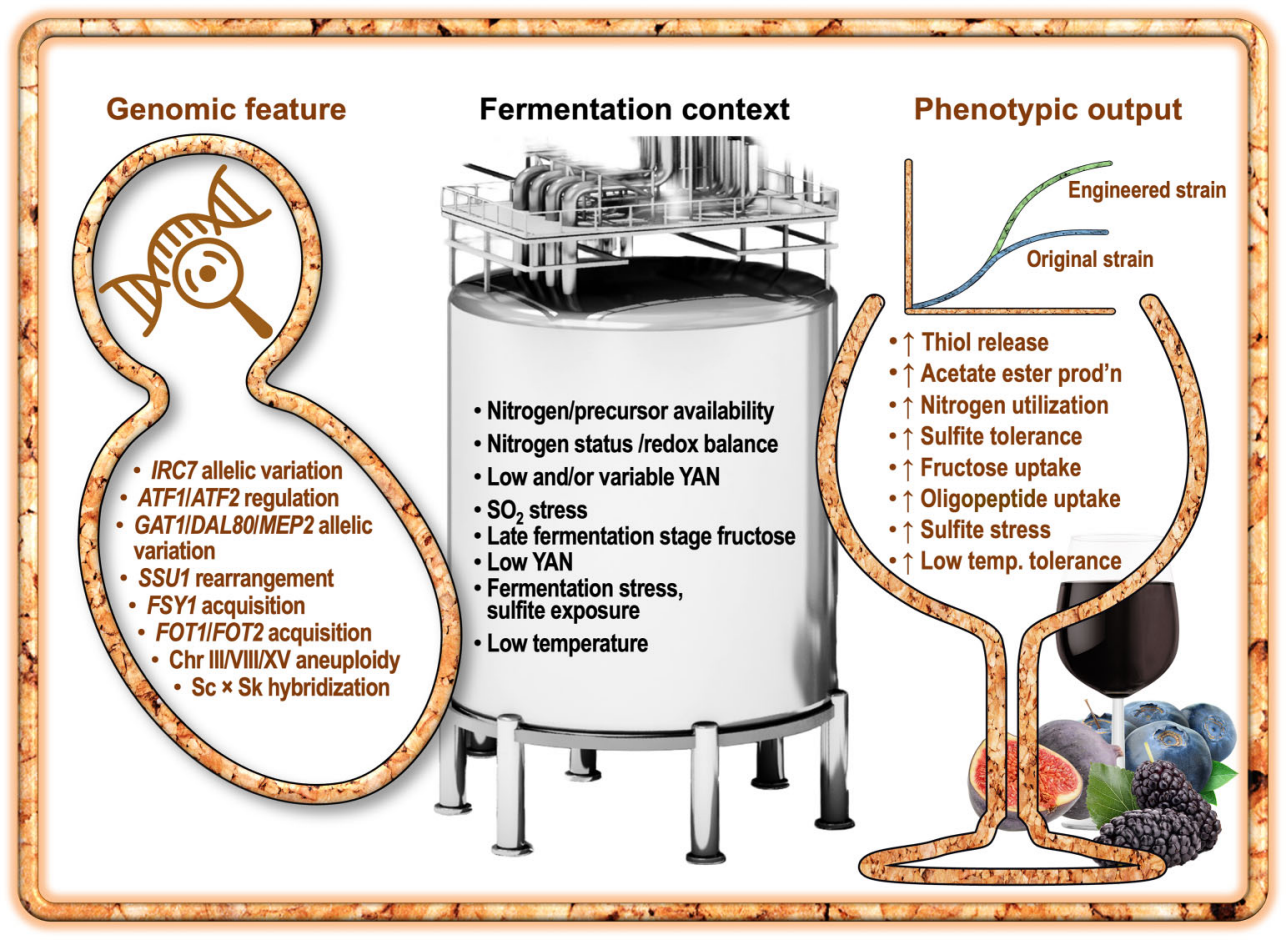
From genotype to phenotype in wine fermentation: a context-dependent framework. Schematic overview of how specific genomic features in wine yeasts interact with fermentation conditions to shape phenotypic outcomes. Genetic variants and structural changes, including *IRC7* allelic variation, *ATF1/ATF2* regulation, *GAT1/DAL80/MEP2* allelic variation, *SSU1* rearrangements, *FSY1* and *FOT1/FOT2* acquisition, chromosome III/VIII/XV aneuploidy, and *Saccharomyces cerevisiae* × *Saccharomyces kudriavzevii* hybridization, are shown in the context of nitrogen availability, redox balance, sulphite stress, late-fermentation fructose conditions, and low temperature. These genotype-by-environment interactions contribute to phenotypic outputs relevant to winemaking, including increased thiol release, acetate ester production, nitrogen utilization, sulphite tolerance, fructose uptake, oligopeptide uptake, and low-temperature tolerance.

Overall, QTL mapping, transcriptomics, proteomics, and systems level analyses reveal the complex, multi-layered genetic and regulatory basis of fermentation performance. They also highlight the difficulty of predicting strain behaviour in variable must environments, underscoring the need for integrative genomic–regulatory–metabolic models to move toward more predictive understanding of fermentation outcomes. Examples of genotype-by-environment relationships shaping fermentation relevant phenotypes in wine yeasts are summarized in Fig. 3.

### Expanding beyond *Saccharomyces*: Non-*Saccharomyces* yeasts and spoilage organisms

High-throughput genomics has shown that non*-Saccharomyces* yeasts bring distinct metabolic and ecological capabilities to wine fermentation, from enhanced glycerol production and lactic acid formation to biocontrol through pulcherriminic acid. Spoilage species, such as *Brettanomyces bruxellensis*, exhibit remarkable genomic plasticity that supports long term survival and the formation of volatile phenols. Integrating these genomic features into strain selection and mixed culture design is now seen as a cornerstone of what has been termed ‘precision oenology’.

#### Beneficial non-*Saccharomyces* species

Despite their growing use in wine biotechnology, non-*Saccharomyces* yeasts remain unevenly characterized compared with *S. cerevisiae*. This is particularly evident for grape associated genera such as *Hanseniaspora*, which are often abundant during early fermentation but can be difficult to phenotype reproducibly under winemaking conditions because of strong strain level variation, sensitivity to must composition, ethanol and sulfite tolerance limits, variable persistence during fermentation, and dependence on co-culture interactions with *S. cerevisiae* and other microbes (van Wyk et al. 2024). Integrated genomic, transcriptomic, and flavour phenotyping studies are therefore especially valuable for linking non-*Saccharomyces* diversity to wine relevant traits. For example, work on *Hanseniaspora vineae* showed that this species possesses and expresses metabolic pathways associated with acetate ester, ethyl ester, higher alcohol, and benzenoid compound formation during fermentation, providing a clear example of flavour impacting functions that differ from, or are absent in, *Saccharomyces* but can operate under industrially relevant conditions (Giorello et al. 2019).

Genomic and functional studies have revealed that many species used in sequential or co-inoculated fermentations possess gene sets that directly contribute to desirable oenological outcomes. *Torulaspora delbrueckii* shows expansions in genes related to stress response, carbohydrate metabolism, and glycerol biosynthesis, which aligns with its observed effects, including reduced volatile acidity, higher glycerol levels, improved mouthfeel, and modestly lower ethanol when co-fermented with *S. cerevisiae* (Varela and Borneman 2017, Benito 2018). *Metschnikowia pulcherrima* carries a pulcherriminic acid (PUL) gene cluster and strong iron scavenging pathways that confer robust biocontrol activity, along with glycosidases capable of releasing terpenes and thiols from glycosylated precursors (Gore-Lloyd et al. 2019, Vicente et al. 2020). *Lachancea thermotolerans* stands out for its expanded lactate dehydrogenase families and associated regulatory elements, enabling significant lactic acid production, a valuable biological tool for must acidification, especially relevant in warmer vintages (Benito 2018, Sizzano et al. 2024). Species of *Hanseniaspora*, such as *H. uvarum*, exhibit genome reduction and signs of accelerated evolution (possibly linked to weakened DNA repair pathways), yet they contribute disproportionately to ester and thiol formation, producing distinctive, strain specific aromatic profiles (Onetto et al. 2025a, Hittinger et al. 2018). These genomic insights support the vision of ‘precision oenology’: deliberately selecting and combining non-*Saccharomyces* species to achieve targeted sensory and technological outcomes in a predictable way (Jolly et al. 2014, Pretorius 2020).

In addition to fermentation performance and aroma formation, non-*Saccharomyces* yeasts differ in downstream cellar relevant traits such as flocculation, sedimentation behaviour, cell death, autolysis, and mannoprotein release. These traits influence clarification, filtration requirements, lees ageing, wine body, mouthfeel, foam properties, protein and tartaric stability, and sensory complexity, particularly in white and sparkling wines. In *S. cerevisiae*, flocculation is strongly associated with cell wall architecture and *FLO* family adhesins, while autolysis releases mannoproteins, polysaccharides, peptides, amino acids, lipids, and other cell derived compounds during ageing on lees. Because *S. cerevisiae* autolysis often requires prolonged ageing, faster lysis or compound release kinetics in some non-*Saccharomyces* species may offer practical advantages (Machado dos Santos and Kempka 2026). Recent comparative work further indicates that mannoproteins from *S. cerevisiae* and non-*Saccharomyces* yeasts can differ in polysaccharide structure and physicochemical properties, making species- and strain-level diversity relevant for wine stability, texture, and lees management (Assunção Bicca et al. 2025).

#### Spoilage yeasts and genomic plasticity

In contrast, spoilage organisms illustrate genomic strategies built around resistance to harsh environments and off-flavour production. Populations of *Brettanomyces bruxellensis* display variable ploidy (diploid to triploid), extensive copy number variation at sulfite resistance loci, and expansions of phenolic acid decarboxylase (PAD) and vinylphenol reductase (VPR) gene families. These adaptations allow survival in nutrient poor, high ethanol, sulfite treated environments and drive the formation of 4-ethylphenol and 4-ethylguaiacol, the classic ‘Brett’ characters (Avramova et al. 2018, Gounot et al. 2020). Other spoilage taxa, such as *Zygosaccharomyces bailii*, carry expanded transporter repertoires (e.g. multi-drug resistance transporters like *ZBA1*), fructophilic metabolism, and weak acid resistance mechanisms, including enhanced acetic acid tolerance via acetate efflux pumps and osmotic stress responses that enable them to persist under preservative conditions and cause refermentation or off-flavours in bottled wines (Mira et al. 2014). Similarly, certain *Starmerella*/*Candida* species exhibit genomic duplications in sugar transporters and acid tolerance genes, facilitating growth in high sugar, low pH conditions and contributing to unwanted ethyl acetate production (Lemos Junior et al. 2018, Raymond Eder and Rosa 2021).

#### Oenological interactions and implications for fermentation design

Genomic and transcriptomic studies have shown that non-*Saccharomyces* yeasts do not simply grow alongside *S. cerevisiae*, but rather actively modulate its physiology through nutrient competition, oxygen consumption patterns, antimicrobial metabolites, and cell–cell signalling and/or interactions. These interactions induce distinct transcriptional responses in *S. cerevisiae*, altering aroma biosynthesis, stress tolerance, and fermentation kinetics (Marsit and Dequin 2015, Varela and Borneman 2017). According to Pourcelot et al. (2023), interactions between wine yeasts during early fermentation are largely species-dependent, while intraspecific variation mainly affects interaction intensity. From a practical perspective, these findings argue for combining population genomics, targeted functional characterization, and systems level modelling to better match strain specific genomic features to particular must compositions and desired sensory profiles. Doing so transforms microbial biodiversity into reproducible, predictable mixed culture strategies, a key direction for future wine yeast research (Hittinger et al. 2018, Pretorius 2020).

## Linking genotype to phenotype: challenges and opportunities

### Polygenic architecture and environment dependent expression

The picture becomes even more complicated because the environment strongly shapes how these genotypes are expressed. Must composition, particularly nitrogen and sugar levels, temperature, oxygen availability, and the presence of other microbes all influence transcription, metabolic fluxes, and fermentation kinetics, often producing quite different phenotypes from the same genetic background. For example, major transcriptional reprogramming occurs in response to the cumulative stresses of wine fermentation (Marks et al. 2008), while nitrogen availability interacts with strain genotype to affect fermentation rate and, indirectly, metabolic pathways linked to aroma precursor formation (Brice et al. 2014). Wine yeast strains exhibit significant variation in their inherent ability to consume nitrogen under oenological conditions, even with excess nitrogen to avoid limitation effects, with wine-adapted lineages showing stronger consumption capacity. This, in turn, enhances overall fermentative robustness and adaptability to wine environments (Brice et al. 2018).

Temperature and nutrient limitation further drive adaptive changes in gene expression, growth behaviour, and nitrogen consumption patterns, especially under the low temperature conditions typical of many white wine fermentations (Garcia-Rios and Guillamon 2019, Garcia-Rios et al. 2024), and the composition and dynamics of the fungal microbiome can markedly alter metabolic outputs and fermentation trajectories even within the same must. Recent large-scale surveys revealed consistent early dominance by *Hanseniaspora uvarum* (often alongside *S. cerevisiae*), together with the variable presence of other genera such as *Starmerella, Metschnikowia*, and *Candida*, and substantial vintage-to-vintage and regional shifts in community structure (Onetto et al. 2024). This early dominance is underpinned by the particularly rapid growth rate of *H. uvarum*, which enables it to quickly shape the fermentation environment before *S. cerevisiae* takes over (Onetto et al. 2025a).

### Gene—environment interactions and the role of the microbiome

Wine musts impose a particularly demanding and constantly changing environment: nitrogen becomes progressively limiting, osmotic pressure is high at the beginning, and redox conditions shift dramatically as fermentation proceeds. These dynamic stresses alone already complicate gene expression and metabolic behaviour. On top of this, structural variation, such as copy number changes, aneuploidies, and introgressed genomic regions, frequently modifies ‘gene dosage’ and regulatory balance. These effects are often highly context dependent, which make them difficult to detect or predict reliably using SNP-based approaches alone (Dunn et al. 2012, Peter et al. 2018).

The microbial community introduces yet another important layer of complexity. Non-*Saccharomyces* yeasts and lactic acid bacteria compete directly with *S. cerevisiae* for nitrogen, influence the redox environment through their own metabolic activity, and modify the pool of aroma precursors available in the must. These interactions give rise to emergent metabolic outcomes, including altered ester synthesis, thiol release and higher alcohol profiles which are very difficult, often impossible, to predict from monoculture studies of *S. cerevisiae* (Hranilovic et al. 2018, Borren and Tian 2020, Onetto et al. 2024, Zhang et al. 2025). Taken together, these observations make a compelling case that any serious attempt to model genotype–phenotype relationships in wine fermentation must incorporate ecological context, both the abiotic stresses of the must and the biotic interactions within the microbial community.

### Systems biology approaches to predict fermentation outcomes

To make headway on these complex challenges, researchers are increasingly adopting systems level approaches that integrate multiple layers of data. By combining genomic variation with transcriptomic profiles, metabolomic snapshots and detailed fermentation kinetic measurements, it is possible to build a much more complete model of what drives fermentation performance. Genome-scale metabolic models, dynamic flux balance analysis and a growing range of machine learning methods are now beginning to uncover how differences in regulatory networks and pathway architecture shape key fermentation outcomes (Barbosa et al. 2022, Pretorius 2022). These tools are particularly useful for capturing non-linear relationships and interactions that traditional reductionist approaches tend to miss.

Researchers have long argued that synthetic genomics, when combined with systems-level thinking, opens a realistic path forward. Rather than just trying to predict behaviour from existing genotypes, these approaches aim to actively understand and eventually redesign complex, polygenic networks in a more controlled and predictable way (Pretorius 2017a). This conceptual framework has become increasingly influential and is now guiding much of the thinking about the next generation of wine yeast strain development.

However, despite these advances, robust genotype—phenotype prediction in wine yeast remains unresolved. Many fermentation traits are not controlled by single variants with stable effects, but arise from polygenic architectures whose expression depends on must composition, temperature, oxygen availability, nutrient status, and microbial context. SNP-based association approaches may also miss important contributions from structural variants, introgressed regions, copy number variation, and aneuploidy, all of which can alter gene dosage and network behaviour. As a result, current models can identify associations and improve mechanistic interpretation, but they rarely provide robust predictions across different musts, vintages, strain backgrounds, and fermentation regimes. This distinction is important, as identifying genetic contributors to fermentation traits does not yet mean that fermentation outcomes can be reliably forecast or engineered under commercial conditions. Addressing this limitation will require controlled fermentations, community aware models, longitudinal multi-omics, and validation across diverse production contexts.

### Case studies: nitrogen metabolism and volatile compound production

Looking at nitrogen metabolism and aroma compound formation gives a clearer sense of both the difficulties and the real potential of integrative approaches. Allelic variation in key nitrogen regulators (*GAT1, DAL80*) and permeases (*MEP2*) interacts strongly with nitrogen availability in the must, influencing fermentation speed, biomass formation, and the flux toward aroma precursors (Beltran et al. 2004, Crepin et al. 2012, Peltier et al. 2019). Higher alcohols, acetate esters and varietal thiols all arise from a combination of coding polymorphisms (e.g. *ATF1*/*ATF2, ARO10, IRC7*), regulatory differences, and environmental conditions, particularly nitrogen status, temperature and redox balance.

Genetic variation in ester forming enzymes and upstream metabolic steps can strongly shift aroma profiles, with clear multilocus effects that are heavily modulated by the environment (Cordente et al. 2012). Recent studies have also shown that thiol release depends not only on variation in *IRC7* and associated β-lyase pathways (Cordente et al. 2019), but also on regulatory responses shaped by must composition and microbial interactions (Thibon et al. 2008, Seguinot et al. 2020, Cordente et al. 2022). Multi-omics approaches that combine genomics, transcriptomics, and metabolomics are beginning to map these relationships more precisely, revealing how flux control, gene dosage and external signals converge to determine final aroma composition (de Celis et al. 2024, Garcia-Rios et al. 2024). These examples illustrate, at trait level, why genotype–phenotype relationships in wine yeasts are often conditional rather than fixed: the same allelic variant may have different effects depending on nitrogen status, temperature, redox balance, and microbial context. Representative genotype–by–environment relationships, including those involving nitrogen assimilation, volatile compound release, and other fermentation relevant phenotypes, are summarized in Fig. 3.

## Translational applications: from genomics to future winemaking innovation

### Rational development of starter cultures

Expanding genomic and multi-omics resources, including population genomics, pan-genomes, and fermentation scale transcriptomic and metabolomic surveys, is shifting starter culture development from empirical selection toward truly data driven design (Dunn et al. 2012, Peter et al. 2018, de Celis et al. 2024). Comparative and systems level frameworks now link genotype to fermentation traits, while regional surveys of wine associated lineages, including Iberian/Mediterranean clades, allow resolution of domestication origins, lineage structure, and accessory genome variation underlying niche adaptations and fermentation performance (Almeida et al. 2015, Lu et al. 2019). Multi-condition transcriptomic, proteomic, and metabolomic profiling across strains further resolves regulatory programs for nitrogen assimilation, stress tolerance, and aroma precursor conversion, while also indicating that predictive performance remains context dependent (Beltran et al. 2004, García-Ríos et al. 2014, de Celis et al. 2024). Machine learning models trained on genotype—phenotype and process-level data increasingly anticipate strain behaviour under must specific constraints, including climate linked shifts in sugar content, acidity, and overall nutrient availability (de Celis et al. 2024, Khaiwal et al. 2025, Watcharawipas et al. 2025). Incorporating pan-genomic features (e.g. introgressions, copy number variants, and structural variants) can lead to strains with improved prediction of fermentation kinetics and aroma trajectories (Peter et al. 2018, de Celis et al. 2024). In parallel, breeding and hybridization approaches already deliver targeted phenotypes, including enhanced stress robustness and, in some cases, reduced ethanol yield with limited sensory faults (Pérez et al. 2022, Kessi-Perez et al. 2025). Starter culture design should also consider downstream cellar-relevant traits, including flocculation, sedimentation behaviour, autolysis, and mannoprotein release, as genomic variation in cell-wall architecture, FLO-family adhesins, and lysis-related pathways may influence clarification, filtration requirements, protein and tartaric stability, lees ageing, mouthfeel, and sparkling or white wine quality (Assunção Bicca et al. 2025, Machado dos Santos and Kempka 2026). Together, these advances define a translational framework for sustainable, precision starter culture design.

### Biotechnological interventions and adaptive strategies

Various biotechnological tools are increasingly applied to traits that are difficult to optimize through standing variation alone. In wine yeasts, CRISPR/Cas9 provides a practical platform to perturb pathways conferring nitrogen catabolite utilization, osmoadaptation and sulfur-associated aroma metabolism (Vigentini et al. 2017, Muysson et al. 2019, Granuzzo et al. 2026). Complementary CRISPR interference (CRISPRi) and genome-wide screening approaches now enable systematic functional interrogation in *S. cerevisiae*, including identification of determinants of stress resilience and acetic acid tolerance (McGlincy et al. 2021, Mukherjee et al. 2021, Robertson et al. 2025). Moreover, targeted editing can also enhance specific quality traits while preserving core fermentation performance; notably, *IRC7* focused cisgenesis increases volatile thiol release from grape-derived precursors (Granuzzo et al. 2026). As a result, such strategies help disentangle causal loci from linked background effects under oenological conditions by enabling controlled perturbation of regulatory nodes. Therefore, designer genome editing represents a precise genotype-to-phenotype framework for wine yeast improvement.

Adaptive laboratory evolution (ALE) approaches complement precision genome editing by selecting beneficial multi-locus architectures under process relevant stress and capturing epistatic or regulatory effects that are difficult to design directly. ALE-derived lines often improve fermentation kinetics and ethanol stress robustness (Novo et al. 2014), or metabolite production (Gardner et al. 2023) consistent with systems level rewiring. Hybridization within the *Saccharomyces sensu stricto* complex offers a parallel route for trait stacking; cryotolerance and related stress traits from non-*cerevisiae* parents can be combined with the fermentative strength of wine *S. cerevisiae*, and both natural and laboratory-generated hybrids perform well in grape must (Gonzalez et al. 2007, Gangl et al. 2009, Garcia-Rios and Guillamon 2019). Multi-round breeding has likewise produced lower ethanol strains with acceptable sensory profiles in pilot fermentations (Kessi-Perez et al. 2025). Overall, biotechnology now enables more mechanistic navigation of the polygenic, environment-dependent basis of wine yeast performance.

### Pathway engineering and synthetic enzyme fusions

Pathway engineering offers modular control over aroma formation, redox balance, and carbon flux in ways that exceed the reach of classical breeding. In *S. cerevisiae*, promoter engineering and targeted overexpression show that acetate ester synthesis scales with alcohol acetyltransferase dosage, particularly *ATF1*, and with acetyl-CoA supply, while elevated esterase activity can buffer excess ester accumulation during fermentation and ageing (Lilly et al. 2006, Cordente et al. 2012). Refactoring of Ehrlich pathway modules further demonstrates that rewiring amino acid catabolism, across aminotransferase, decarboxylase and dehydrogenase steps, can redirect higher alcohol fluxes and rebalance by-product formation (Kondo et al. 2012).

The Sc2.0 project extends this paradigm from pathway-level intervention to chromosome and genome-scale design. Synthetic genome principles established in the Sc2.0 framework (Richardson et al. 2017, Pretorius and Boeke 2018), together with SCRaMbLE-based diversification, enable exploration of large structural variant landscapes and discovery of genotype combinations that may be difficult to obtain through conventional crossing (Blount et al. 2018, Jia et al. 2018, Kutyna et al. 2022). More broadly, structural rearrangement driven genome evolution provides both the conceptual rationale and the technical foundation for directed, genome scale optimization, as demonstrated by emerging recombination-based engineering systems (Zhou et al. 2021), while emerging *de novo* genome design frameworks highly support the transition toward custom design/programmable microbial chassis (Koster et al. 2026).

In parallel, engineered or rationally assembled consortia has been long proposed to steer fermentation trajectories through reproducible interspecies interactions, providing a complementary route to single strain pathway engineering and enabling metabolic outcomes that are difficult to achieve with *S. cerevisiae* alone (Ciani et al. 2010). Overall, pathway engineering and synthetic genome approaches are converging toward a mechanistic design framework for fermentation, linking modular pathway control with genome scale predictability, programmability, and subsequent design, to achieve precise outcomes.

### Synthetic pan-genomics and predictive models for fermentation performance

Wine yeast pan-genomic resources show that fermentation phenotypes are shaped by far more than core SNP variation. Large-scale comparisons across *S. cerevisiae* populations reveal extensive structural variation, introgressed segments, copy number polymorphisms, and accessory loci that track with lineage history and technological adaptation (Almeida et al. 2015, Peter et al. 2018, Loegler et al. 2024). In wine populations, these architecture-level features repeatedly associate with production relevant traits, including nitrogen use efficiency, stress robustness and aroma related metabolism, supporting their use as predictive variables rather than only subsequent annotations (Marsit and Dequin 2015, Marsit et al. 2015, Legras et al. 2018). Synthetic pan-genome engineering further expands this design space by experimentally assembling non-core diversity at chromosome-scale, enabling controlled evaluation of accessory gene contributions to fermentation traits (Kutyna et al. 2022, Goold

et al. 2025).

Genome-scale metabolic models and related constraint-based frameworks increasingly formalize genotype-to-phenotype hypotheses for fermentation systems (Bordbar et al. 2014, Sanchez et al. 2017, Lu et al. 2019). These approaches estimate condition-dependent flux reallocations and physiological trade-offs and, when coupled with process-level and multi-omics data, provide quantitative scaffolds for predicting fermentation kinetics, nitrogen consumption dynamics, and by-product trajectories. Machine learning layers built on top of genomic and systems-level features capture nonlinear interactions that purely mechanistic models may miss, making hybrid predictors attractive for strain selection and process tuning (Harrison et al. 2024, Khaiwal et al. 2025, Watcharawipas et al. 2025). As climate-driven must profiles shift toward higher sugar loads and more variable assimilable nitrogen, integrating pan-genomic architecture with GEM-based and data-driven models is becoming a practical route to anticipatory, design-oriented winemaking.

### Designing sensory profiles for targeted consumer markets

Recent advances in yeast genomics, metabolic engineering, and predictive modelling are shifting strain development from broad robustness optimization toward intentional sensory design. Instead of selecting solely for fermentation completion and stress tolerance, current frameworks map molecular variation, allelic diversity, regulatory variants, CNVs, and pathway activity, to explicit style and market targets. Practically, this enables strains tailored for stronger varietal thiol expression in Sauvignon-Blanc-like profiles, moderated volatile acidity and fresher balance in warm climate reds, or enhanced fruity ester signatures for early drinking styles (Swiegers et al. 2005, Lilly et al. 2006, Cordente et al. 2012, Granuzzo et al. 2026).

CRISPR-enabled cisgenesis provides a clear demonstration of causal control; targeted optimization of *IRC7* in oenological backgrounds increases volatile thiol release while maintaining acceptable fermentation behaviour, showing that specific aroma determinants can be tuned without wholesale strain redesign (Granuzzo et al. 2026). More broadly, integrating multi-omics features, like genomics, transcriptomics, and volatile composition profiles, with chemometric and machine learning methods, enables forward prediction of sensory trajectories and consumer relevant attributes (Wold et al. 2001, Barbosa et al. 2022). Although performance remains matrix and process dependent, these tools support a design-oriented paradigm that aligns yeast selection and engineering with product differentiation, sustainability constraints and segmented market demand (Pretorius 2000).

## Future directions and perspectives

### Integration of multi-omics data to better understand the winemaking process

The next phase of wine yeast genomics is likely to be driven by deeper integration of multi-omics datasets, such as genomics, transcriptomics, proteomics, metabolomics, and fluxomics, collected under process-relevant fermentation conditions (Pretorius 2022, de Celis et al. 2024, Qin et al. 2025, Minebois et al. 2025, Moimenta et al. 2025). While individual one-dimensional ‘omics layers have clarified regulatory programs for nitrogen metabolism, stress tolerance, and aroma formation, their combined interpretation remains constrained by a non–standardized sampling frameworks and the dynamic, highly complex nature of wine fermentation (Beltran et al. 2004, Zuzuarregui et al. 2006, Mendes et al. 2017).

Recent studies show the value of this integration. Fermentation time course datasets combining transcriptomics and metabolomics revealed coordinated regulation of amino-acid catabolism, transporter activity, and Ehrlich-pathway-linked aroma outputs, helping explain strain specific volatile profiles (Mendes et al. 2017, Minebois et al. 2021, de Celis et al. 2024). Similarly, transcriptome-proteome analyses show that post–transcriptional regulation and membrane-lipid remodelling are central to stress adaptation, including both ethanol and low temperature tolerance (Zuzuarregui et al. 2006, García-Ríos et al. 2014).

High resolution, time structured multi-omics sampling should improve recognition and reconstruction of regulatory cues, reveal condition-specific bottlenecks, and support quantitative models linking molecular variation to fermentation outcomes with greater accuracy. Such datasets are also essential for separating strain specific regulatory architecture from environmental effects, a prerequisite for robust genotype-phenotype prediction across various winemaking contexts (Minebois et al. 2021, de Celis et al. 2024).

### Ecological genomics: vineyard-to-cellar microbial interactions

Wine fermentation is an inherently ecological process. Grapes arrive at the winery colonized by complex microbial communities shaped by vineyard biogeography, climate, soil, and management practices. These communities undergo rapid succession during crushing, must preparation, and alcoholic fermentation, generating continually changing interspecies interactions that influence fermentation kinetics, metabolite profiles, and sensory outcomes (Bokulich et al. 2016, Griggs et al. 2021, Griggs et al. 2025).

Advances in ecological genomics, integrating multi–omics approaches with vineyard–to–cellar microbial profiling, now enable increasingly detailed mapping of how grape associated communities evolve during processing (de Celis et al. 2024, Griggs et al. 2025). Culture independent microbial surveys reveal strong regional signatures in grape and must microbiota, shaped by environmental factors and vineyard practices (Bokulich et al. 2016, Griggs et al. 2021). These microbial communities are not passive, but actively shape spontaneous fermentation, with early non–*Saccharomyces* taxa such as *Hanseniaspora, Lachancea*, and *Metschnikowia* influencing metabolite formation, environmental conditions, and the timing of *Saccharomyces* takeover. These early interactions depend on multiple process parameters, including SO₂, must sugar concentration, acidity, and pH, and contribute to downstream differences in aroma composition and fermentation progression (Bokulich et al. 2016, Borren and Tian 2020, Onetto et al. 2023, 2025b). Multi–omics studies increasingly connect community-level patterns to underlying genomic and transcriptomic mechanisms, showing that dominant strain or species composition can account for substantial variance in fermentation metabolite profiles. Ortholog– and pathway–level analyses further reveal species-specific regulation of amino acid transport, sulfur metabolism, and Ehrlich pathway functions that contribute directly to aroma formation (Mendes et al. 2017, Minebois et al. 2021, de Celis et al. 2024).

This perspective also underpins the microbial basis of ‘terroir’; regionally distinct grape and must associated communities are linked to distinct volatile and metabolite profiles even under partially standardized fermentation conditions, supporting a measurable microbial contribution to regional wine identity (Griggs et al. 2021, Minebois et al. 2021). Looking forward, ecological genomics is increasingly converging with synthetic biology frameworks. Advances in genome-scale and enzyme-constrained metabolic modelling, together with biodiversity aware predictive engineering, point toward the rational design of yeast consortia and community-scale models capable of dissecting higher order interactions and enabling stable, predictable fermentations tailored to specific winemaking contexts (Sanchez et al. 2017, Lu et al. 2019, Watcharawipas et al. 2025).

### Predictive modelling and AI—enabled fermentation management

Machine learning and hybrid mechanistic-statistical models are rapidly becoming practical tools for predictive, design-oriented winemaking. As genomic and pan-genomic variation, including structural variants and accessory loci, is integrated with multi-omics and process-level measurements, these models increasingly capture strain and condition dependent differences in fermentation kinetics, nitrogen utilization, and aroma-linked metabolic trajectories (Henriques et al. 2021, de Celis et al. 2024, Khaiwal et al. 2025).

Recent studies illustrate this pattern: ML models applied to wine fermentation datasets show strong potential for predicting fermentation dynamics and high-quality outcomes across variable conditions, including varying temperature and nutrient environments, as well as mixed–culture compositions (Balsa-Canto et al. 2020, Barbosa et al. 2022, Sanchez-Roca et al. 2026). In parallel, genome–scale metabolic models provide mechanistic constraints that enhance interpretability and increasingly reliable predictability. Emerging work combining mechanistic modelling with data–driven approaches suggests that hybrid frameworks may offer more robust genotype–to–phenotype predictions than either alone, particularly for complex, fermentation relevant traits (Sanchez et al. 2017, Lu et al. 2019, Watcharawipas et al. 2025).

**Figure 4:**
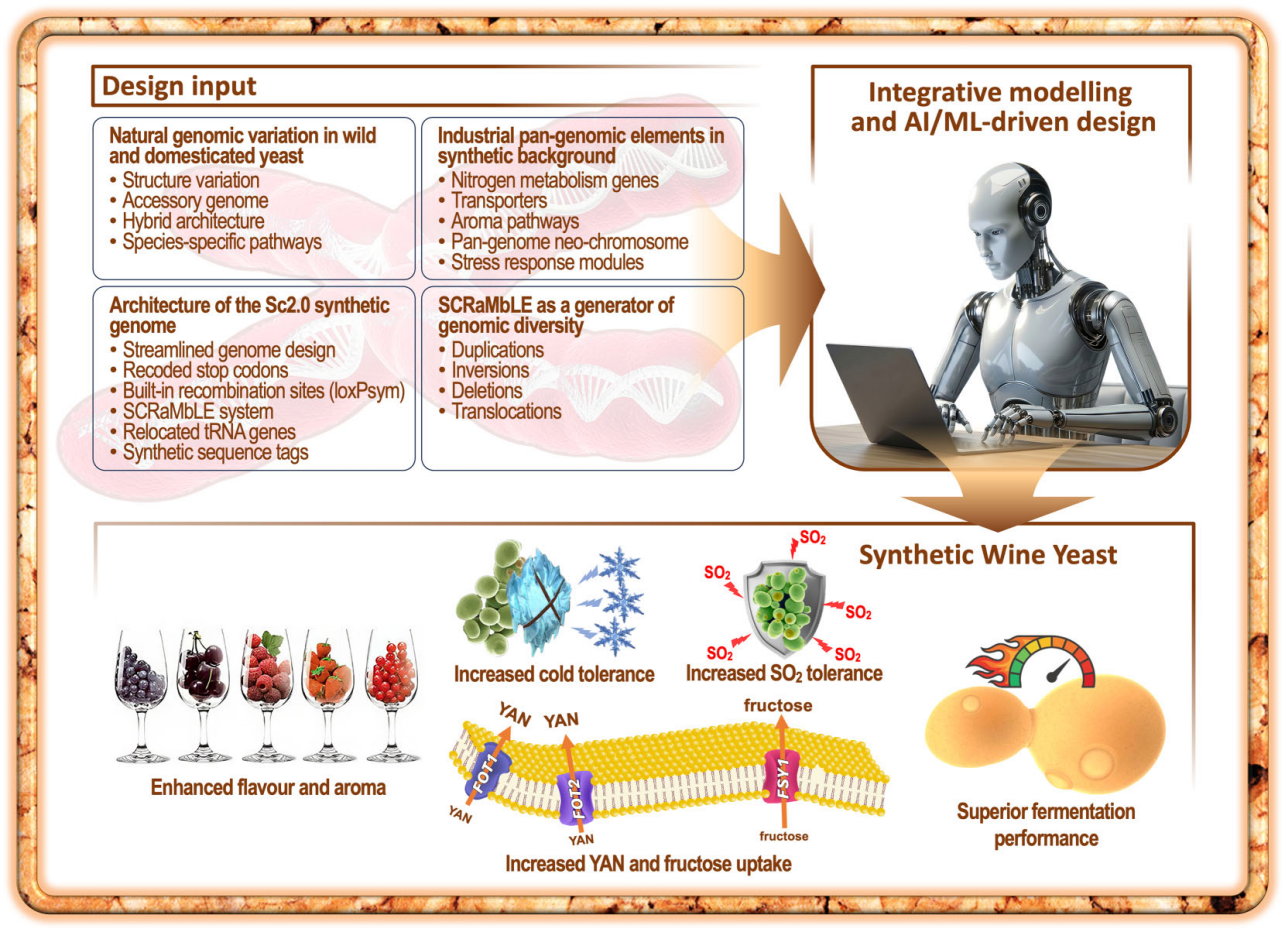
AI- and ML-enabled routes to modern wine yeast strain design. Schematic overview of a next-generation framework for engineering synthetic wine yeasts by integrating natural diversity, pan-genomic content, and synthetic genome design with artificial intelligence and machine learning. Design inputs include natural genomic variation in wild and domesticated yeasts, industrial pan-genomic elements introduced into synthetic backgrounds, features of the Sc2.0 synthetic genome platform, and SCRaMbLE-generated genomic diversity through duplications, inversions, deletions, and translocations. These inputs are funnelled through integrative modelling and AI/ML-driven design to generate synthetic wine yeasts with superior fermentation performance, enhanced yeast assimilable nitrogen (YAN) and fructose uptake, increased SO₂ and cold tolerance, and enhanced flavour and aroma production.

The convergence of these approaches offers a realistic path toward data–driven decision support for strain selection, nutrient management, and temperature control tailored to grape must composition and target sensory profiles. Building on recent advances in predictive modelling of fermentation dynamics, AI–assisted optimization may eventually support semi–autonomous or closed loop fermentation management, where model outputs iteratively adjust fermentation parameters to stabilize desired trajectories despite variability in grape must composition (Barbosa et al. 2022, de Celis et al. 2024, Sanchez-Roca et al. 2026). Despite this progress, the current contribution of multi-omics and machine learning should be viewed primarily as improved interpretation and condition specific prediction, rather than universal forecasting of wine fermentation outcomes. Multi-omics datasets can now identify regulatory, metabolic, and ecological factors associated with fermentation kinetics, nitrogen use, stress adaptation, and aroma production, while ML models can detect nonlinear patterns within well defined experimental or process datasets. However, their broader predictive power remains limited by non-standardized sampling, uneven metadata quality, limited strain and vintage coverage, matrix dependent sensory outcomes, and incomplete validation under realistic winery conditions. Thus, fully transferable, AI-enabled fermentation management remains a prospective goal rather than a routine current capability. An AI/ML-enabled framework for integrating genomic design inputs, synthetic genome engineering, and predictive modelling in next-generation wine yeast development is summarized in Fig. 4.

### Towards reliable genotype—phenotype prediction for wine quality

Reliable genotype—phenotype prediction remains a central goal for wine yeast research, but future progress will depend less on generating more data alone and more on validating whether models remain accurate across independent strains, must compositions, vintages, microbial communities, and production scales. This requires integration of high resolution genomic and pan-genomic variation, multi-omics and systems biology frameworks, and ecological or process level measurements that contextualize strain behaviour during fermentation (Giaever et al. 2002, Peter et al. 2018, Lu et al. 2019). Comparative and pan-genomic studies already show that accessory loci, introgressions, and structural variation contribute to fermentation relevant phenotypes, while multi-omics informed and constraint based models improve interpretation of strain specific outputs under changing fermentation conditions (Ambroset et al. 2011, Marsit and Dequin 2015, Sanchez et al. 2017, Peter et al. 2018, Lu et al. 2019). Ecological genomics further shows that vineyard and cellar derived microbial communities can modulate realized phenotypes beyond what single strain genotypes predict (Onetto et al. 2025a, Bokulich et al. 2016, Borren and Tian 2020, Griggs et al. 2025).

The priority now is to convert these insights into standardized, transferable, and experimentally validated prediction frameworks. Such frameworks should be evaluated not only by their ability to explain past fermentation outcomes, but also by their usefulness for practical decisions including strain selection, nutrient management, sensory profile design, climate adaptation, and lower intervention winemaking (Pretorius 2017b, 2022). Overall, wine yeast genomics is moving from descriptive cataloguing toward predictive and design oriented oenology, but this transition will depend on benchmarking datasets, mechanistically interpretable models, ecological validation, and realistic winery scale testing.

Several priorities emerge for the next phase of wine yeast genomics. First, predictive models need standardized, longitudinal datasets that include genomic, multi-omics, fermentation kinetic, chemical, sensory, and metadata layers collected across diverse strains, musts, vintages, and production scales. Second, genotype—phenotype models should be benchmarked across independent datasets to test transferability rather than only retrospective fit. Third, ecological context must be incorporated explicitly, including non-*Saccharomyces* yeasts, bacteria, and vineyard-to-cellar community succession. Finally, promising predictions should be validated experimentally under realistic winery conditions, so that precision oenology moves from conceptual potential toward robust, operational decision making.
